# Significance and Role of Pattern Recognition Receptors in Malignancy

**DOI:** 10.1007/s00005-019-00540-x

**Published:** 2019-04-11

**Authors:** Jan Żeromski, Mariusz Kaczmarek, Maciej Boruczkowski, Agata Kierepa, Arleta Kowala-Piaskowska, Iwona Mozer-Lisewska

**Affiliations:** 1 Department of Clinical Immunology, Karol Marcinkowski University of Medical Sciences, Poznań, Poland; 2 Department of Infectious Diseases, Hepatology and Acquired Immunodeficiencies, Karol Marcinkowski University of Medical Sciences, Poznań, Poland

**Keywords:** PRRs, TLRs, Cancer cells, Agonists, Nucleic-acid sensing, RLRs, cGAS-STING pathway

## Abstract

Pattern recognition receptors (PRRs) are members of innate immunity, playing pivotal role in several immunological reactions. They are known to act as a bridge between innate and adaptive immunity. They are expressed on several normal cell types but have been shown with increasing frequency on/in tumor cells. Significance of this phenomenon is largely unknown, but it has been shown by several authors that they, predominantly Toll-like receptors (TLRs), act in the interest of tumor, by promotion of its growth and spreading. Preparation of artificial of TLRs ligands (agonists) paved the way to use them as a therapeutic agents for cancer, so far in a limited scale. Agonists may be combined with conventional anti-cancer modalities with apparently promising results. PRRs recognizing nucleic acids such as RIG-1 like receptors (sensing RNA) and STING (sensing DNA) constitute a novel promising approach for cancer immunotherapy.

## Introduction

Pattern recognition receptors (PRRs) are protein molecules localized on cell surface or in intracellular space in almost all members of animal kingdom, predominantly in *Eukariota.* They are factors of innate immunity. Their task is to recognize both, external hazards such as various microbes and internal ones as noxious products of metabolism, remnants of dead or dying cells and other. They are old evolutionary receptors present in practically all vertebrates investigated so far, some invertebrates and their homologues in plants. In the case of pathogenic microorganisms, PRRs are able to recognize various organic compounds pivotal for growth, development, and proliferation of microbes, such as lipopolysaccharides, complex lipids, various carbohydrates, nucleic acids etc. They are designated as pathogen-associated molecules (PAMPs). Other substances, products of host’s own metabolism including uric acid, bile acid salts, remnants of cell death, some minerals and other are known as danger (or damage)-associated molecular patterns (DAMPs). There are several families of PRRs. The latter are further subdivided into individual sensors according to recognized chemical specificity. The best known and as the first detected are Toll-like receptors (TLRs), showing some ten items in humans. Other PRRs include NOD-like receptors (NLRs), C-type lectin ones and RIG-1 like receptors (RLRs) (Akira and Takeda 2004; Medzhitov 2013). The last ones are involved in anti-tumor defense.

Another family often linked with PRRs is cytosolic DNA sensors referred as STING (stimulator of interferon genes). The latter are transmembrane proteins residing in endoplasmic reticulum, able, following contact with cytosolic DNA, after translocation to Golgi apparatus, to phosphorylate interferon regulating factor 3 (IRF3) and to induce subsequent production of type I interferon (IFN).

Some PRRs, especially TLRs were shown to participate in malignant phenomena both, in animals and man (Woo et al. 2015). There are several cues to be considered while trying to elucidate the role of these molecules in neoplastic events (Hirsch et al. 2010; Żeromski et al. 2008).

## Expression and Activation of PRRs (TLRs) on Tumor Cells, Tumor-Infiltrated Lymphocytes and Its Biological Effects

**Fig. 1:**
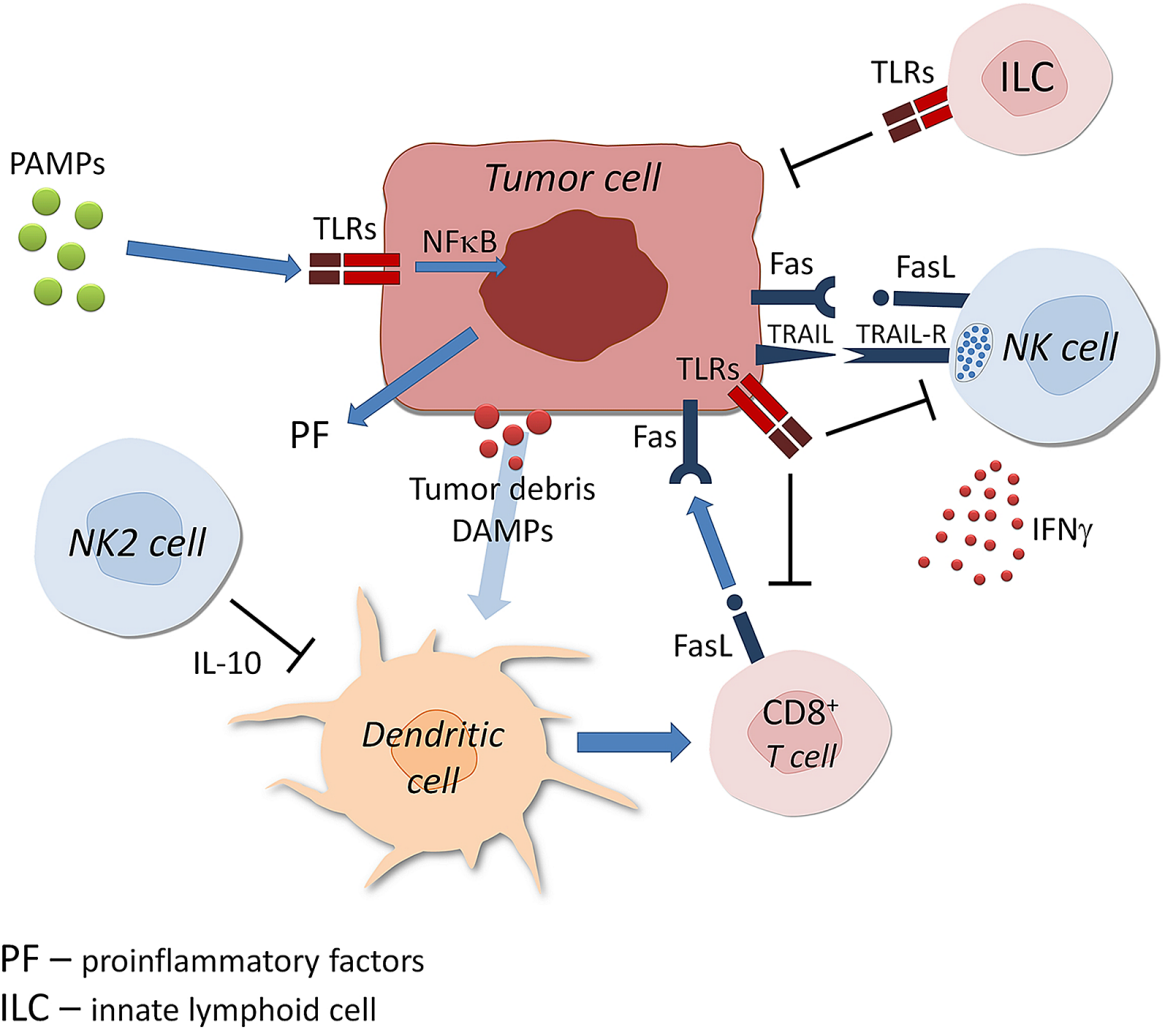
Toll-like receptors—tumor cells: complex interactions

Several PRR molecules have been demonstrated in/on cells of various cancer cells, including lung, head and neck, colon, breast, stomach, ovary, and other (Damasdi et al. 2017; Fukata et al. 2007; Gowing et al. 2017; Ikehata et al. 2018; Jiang et al. 2017; Park et al. 2017; Royse et al. 2017; Yue et al. 2017) (Table 1). Interactions between tumor cells and TLRs are complex. They involve not only sensing of PAMPs of microbial origin, but also reactions with tumor infiltrating cells (TIC) such as NK cells, dendritic cells (DCs), CD8^+^ T cells, innate lymphoid cells and others (Matsumoto et al. 2017). TLRs expressed on TIC become stimulated by DAMPs (tumor debris) leading to their activation such as DCs with subsequent antigen presentation to CD8^+^ T cells and their anti-tumor effect (Fig. 1). In general, however, in the majority of cancers TLRs expression appears to have tumor-promoting effect. Pancreatic cancer cells show expression of TLR2, TLR4 and TLR9, but their functional follow-ups differ. TLR4 activation promotes angiogenesis (Sun et al. 2016b), while high cytoplasmic expression of TLR9 was associated with longer patient survival (Leppänen et al. 2017). Signaling of all three TLRs mentioned promotes autoregulative tumor cell growth and anti-apoptotic Bcl-xL expression (Grimmig et al. 2016; Won et al. 2017). In animal model of prostatic cancer it has been shown that expression of TLR9 on tumor cells has the tumor-propagating potential. The mechanism of tumor progression appears to be the induction of myeloid-derived suppressor cells triggered by leukemia-inhibitory factor secreted by cancer cells. In human epithelial ovarian cancer tested on tissue samples of 500 patients by immunohistochemistry, it was shown that expression of TLR4 and MyD88 predicts poorer overall patient survival apparently due to favoring inflammatory microenvironment at the site of tumor growth (Li et al. 2016). In hepatocellular carcinoma the role of TLR4 expression has been universally accepted in induction of this cancer via several mechanisms including the rise of Treg cells, liver resident follicular helper-like T cells as well as increased formation of pro-inflammatory and malignancy-related molecules (Song et al. 2018). Apart from TLR4, other TLRs as TLR2, TLR3, and TLR9 were already shown, in preceding cancer, hepatic cirrhosis (Sun et al. 2016a; Yin et al. 2017). In hepatocellular carcinoma TLR4 expression was classified as a possible carcinogenic agent, due to its ability to increase quantity of several pro-inflammatory and malignancy-related molecules such as NANOG, Caspase-1 and others (Sepehri et al. 2017). In human papillomavirus (HPV)-positive oropharyngeal cancer, expression of TLR5 and TLR7 correlated with tumor recurrence. High TLR5 and low TLR7 expression were in line with poor disease-specific survival (Jouhi et al. 2017).

**Table 1:** Impact of TLRs expression on growth in various cancers

TLR type	Cancer type	Effect on cancer	References
TLR5, TLR7	HPV, SCC	Tumor recurrence	Jouhi et al. (2017)
TLR1/TLR2, TLR6	Chondrosarcoma	Tumor suppression	Galoian et al. (2018)
TLR2	Oral SCC	Tumor progression	Ikehata et al. (2018)
TLR3, TLR4	Breast	Longer survival	Vacchelli et al. (2016)
TLR9	Pancreatic	Better prognosis	Leppänen et al. (2017)
TLR2, TLR4, TLR9	Pancreatic	Tumor growth promotion	Jiang et al. (2017)
TLR3	Colon	Promotion of metastases	Bugge et al. (2017)
TLR9	Prostate	Tumor evasion	Won et al. (2017)
TLR4	Head and neck	Tumor growth promotion	Szczepanski et al. (2009)

HPV, human papillomavirus; SCC, squamous cell carcinoma

In colorectal cancer TLR4 activation protects tumor cells by preventing their lysis (Huang et al. 2005). It also promotes colon cancer cell survival after chemotherapy (Chung and Kim 2016). TLR4 siRNA was found to inhibit proliferation and invasion of colon cancer cells in experimental mouse model (Ye et al. 2016). On the other hand, TLR3 was promoting invasiveness of metastatic colorectal cancer by induction of pro-inflammatory cytokines (Bugge et al. 2017). On the other hand there is a report that TLR3 can directly induce apoptosis in human tumor cells (Salaun et al. 2006) It is suggested, that TLR signaling is involved in tumor proliferation but not in cancer initiation (Coleman and Haller 2018). In colorectal cancer it is supposed to be due to bacterial microbiome dysbiosis of intestinal tract and aberrant function of NLRs. It was found in animal models that NOD1-deficient T cells exhibit impaired IFN-γ production during experimental inflammation which promotes carcinogenesis (Zhan et al. 2016). It is postulated that nucleic acids sensing other PRRs apart from TLRs such as AIM2-like receptors, retinoic acid-inducible gene-1 (RIG-1)-like ones and others participate in development of colitis and subsequently cancer due to the release inflammatory cytokines and the induction of inflammatory milieu (He et al. 2017). An intrinsic tumor suppressor, an *NLRX1* gene product, was shown in intestinal epithelial cells acting as an inhibitor of key tumor-promoting pathways. *NLRX1-*negative mice had increased susceptibility to colorectal cancer (Lei and Maloy 2016; Koblansky et al. 2016).

We have tested TLRs expression in tissue sections by immunohistochemistry, in five cell lines by flow cytometry and RNA by RT-PCR in human laryngeal squamous cell carcinoma. TLR2 and TLR4 expression predominated while other TLRs were shown occasionally. On the other hand, mRNA of TLRs from 1 to 10 could be demonstrated in all cell lines tested (Sikora et al. 2010; Szczepanski et al. 2007). Finally it was shown by us that TLR4 expressed on tumor cells is the agent promoting growth and preventing cells from cell-mediated immunity in head and neck squamous cell carcinoma (Szczepanski et al. 2009). Expression of TLR4 combined with MyD88 in epithelial ovarian cancer turned out to be independent risk factor for shortened survival time (Li et al. 2016). In gastric cancer TLR2 expression in tumor cells was shown in > 50% patients. It was associated with upregulation of anti-apoptotic molecules such as BCL2 or BIRC3 and predicted poor patient outcomes (Castano-Rodriguez et al. 2014; West et al. 2017). It has been also shown on human cancer cell lines, stimulation of tumor expressed TLR3 results in metabolic reprogramming manifested by aerobic glycolysis, enhanced cell migration, elevation of reactive oxygen species as well as upregulation of various genes involved in cancer progression (Matijevic Glavan et al. 2017). In HPV-positive oropharyngeal squamous cell carcinoma TLR5 and TLR7 expression in cancer cells was associated with poor survival (Jouhi et al. 2017). In cervical cancer TLR4 was found to promote proliferation and apoptosis resistance of tumor cells (Jiang et al. 2017). Thus, it may be concluded, that in the majority of human cancers, expression of TLRs on/in tumor cells is connected with poor prognosis.

## The Role of TLR Agonists on Tumor Cells’ Behavior

Several PRRs ligands may be derived from microbial cells or secretions, but for practical application they suffer from various disadvantages. These natural products are usually undefined structures, often polymeric, and as a rule, unsuitable for manufacturing on a commercial scale. The mode of their action is often complex, unpredictable, and possibly they exhibit serious unwanted effects in living organisms. With the increasing knowledge of structure, and function of natural products and innate receptor biology, researchers managed to synthesize novel simplified substances using tools of modern chemistry, that mimic natural PRRs ligands. Several of them possess properties of immune adjuvants, able to stimulate antigen-presenting cells, when applied topically with antigen as a vaccine (Li et al. 2017a; Mikulandra et al. 2017; Wu 2016). Several synthetic PRR ligands were produced, most of them for TLRs (Wu 2016). The best-known TLR agonists concern endosomal TLR7 and TLR8 recognizing ssRNA. Imidazoquinoline, later called imiquimod, agonist of TLR7 was found to possess antiviral activity, initially shown in animal models and later in humans (Chang et al. 2005; Mauldin et al. 2016). Another TLR7 agonist, 852A was found to stimulate plasmacytoid DCs to produce IFN type I and activate both CD8^+^ T cells and NK cells resulting in anti-tumor response (Inglefield et al. 2008; Weigel et al. 2012). Agonists of TLR9 sense unmetylated CpG dinucleotides (CpG ODN). The latter artificially produced, induce several wanted immune responses such as enhanced innate immunity and adaptive Th1 response (Krieg 2007). Polyinosinic–polycytydylic acid (poly:C) known to be the double-stranded RNA, able to act as the ligand of TLR3, was shown to inhibit growth of radioresistant Lewis lung carcinoma in mice, when applied in concern with radiotherapy (Yoshida et al. 2018). Poly:C given in vivo epicutaneously into human skin resulted in the activation of PRRs in Langerhans cells and keratinocytes manifested by the induction of IFN-regulatory factor 3 and NF-κB (Tajpara et al. 2018). Another DC-targeting TLR ligand, h11c tested in animal models was shown to significantly inhibit of tumor growth, and to extend survival, when applied in combination with IFN-γ and cyclooxygenase inhibitor (de Silva et al. 2017). Novel TLR8 agonist WTX2337 (motolimod) was shown to stimulate not only TLR8 but also NLRP3 inflammasome complex resulting in cytotoxic NK cell activation. Phase I open-label studies on human squamous cell carcinoma of the head and neck are in progress (Dietsch et al. 2016). NK cell cytotoxicity against high-risk neuroblastoma could be strongly enhanced by plasmocytoid dendritic cells activated by TLR9. It results in cell-surface expression of TNF-related apoptosis-inducing ligand and marked IFN-γ production by NK cells (Cordeau et al. 2016). Similar findings related to the inhibition of breast cancer cells in vitro and in vivo in mouse models were shown by Wu et al. (2017a). In another mouse model, lung melanoma metastases were partly removed by NK cells exposed to alveolar macrophages stimulated by TLR9 and TLR3 agonists (Sommariva et al. 2017). Examples of application of TLRs ligands in cancer therapy are shown in Table 2.

**Table 2:** TLRs ligands (agonists) in cancer immunotherapy

TLR type	Cancer type	Agonist applied	References
TLR7/TLR8	Basal cell, other skin cancers	Imiquimod	Chang et al. (2005)
TLR7	Hematologic tumors	852A	Inglefield et al. (2008)
TLR7	Melanoma	852A	Dummer et al. (2008)
TLR9	Myeloma	C792	Ray et al. (2014)
TLR5	Advanced solid tumors	CBLB502 (Entolimod)	Burdelya et al. (2013)
TLR3	Advanced solid tumors	Poly(I:C)	Guo et al. (2012)
TLR4	HBV, cervical, Cervarix vaccines	MPL	Paavonen et al. (2009)

14 TLR ligands (77.8%) from 82 studies have been shown to display anti-tumor property in various cancers (Shi et al. 2016)

HBV, hepatitis B; MPL, monophosphoryl lipid A

There are many novel approaches aiming to improve effectiveness of TLR agonists (Shi et al. 2016). One of them is the conjugation of two or more agonists. Their synergistic action, for example of TLR2 and TLR9 or TLR2 and TLR4 appeared to show higher activity than unconjugated mixtures (Nouri-Shirazi et al. 2018; Tom et al. 2015). Another approach is simultaneous targeting of Toll- and NOD-like receptors (Garaude et al. 2012). Among newly produced TLR agonists a small molecule GS-9620, TLR7 ligand raised much interest because of promising antiviral efficacy in chronic hepadnavirus (HBV) infection. Applied orally, it was shown to be efficient in the treatment of experimental hepatitis B infection in woodchucks as well in chimpanzee and in initial clinical trials of hepatitis B patients (Cane et al. 2015; Rebbapragada et al. 2016). As HBV is known as a major carcinogen responsible for hepatocellular carcinoma, GS-9620 might be possibly used in the future as a preventing agent of this carcinogenesis.

## Combination Therapy Applying PRRs Ligands and Conventional Radio/Chemotherapy

Relatively modest results of cancer therapy applying TLR agonists alone fostered researchers to look for other approaches. It was intelligible, that attention was directed to try linking conventional tools of cancer management, i.e., physical and chemical agents with apparently biologically mild compounds such as TLR agonists. It has been so far tested on animal models. For example, Dewan et al. (2012) applied topical TLR7 agonist in concern with low-dose cyclophosphamide and irradiation in mouse cutaneous breast cancer with apparently promising results. UV irradiation was shown to activate TLR9 in human keratinocytes (Pacini et al. 2017). In mouse models activation by unmetylated CPG ODN synergized with a range of chemotherapy regimens including topotecan, 5-fluorouracil, gemcitabine and others in various mouse tumor models. The mechanism of this synergy possibly may result from depletion of Tregs by chemotherapy (cited by Krieg 2007). Yoshino et al. (2018) found that activation of RIG-1-like receptors (RLRs) by agonists (poly I:C) on human non-small cell lung cancer cells in vitro combined with ionizing radiation resulted in effectively induced, caspase-mediated cell apoptosis. This treatment was far more efficient than above-mentioned regimens used alone.

## Anti-Nucleic Acid-Mediated PRRs Cancer Sensing and Hopes for Possible Therapy

Several cancers have viral etiology and PRRs participate in viral tumorigenicity (Thompson and Iwasaki 2008). In HPV-positive cervical cancer it was shown that dysplastic and carcinomatous epithelial cells have different TLR mRNA levels as compared to normal cells. An increase in TLR3 and a decrease in TLR1 mRNA were found. Moreover, in the disease, progress severity was linked to the increase of TLR1, 2, 5, 6, and 9 mRNA levels (DeCarlo et al. 2012). In other gynecological malignancies such as endometrial and ovarian cancers several differences in the expression of various TLRs were demonstrated. The authors concluded that TLRs are critical immunomodulators that may play an important role in the development of these tumors (Husseinzadeh and Davenport 2014). Epstein Barr virus was shown to stimulate TLR and autophagy-bound pathways and to impair maturation of plasmocytoid dendritic cells what may facilitate viral immune escape in lymphoid malignancies (Severa et al. 2013). In Kaposi’s sarcoma, known to be induced by herpes 8 virus, marked reduction of TLR2 and TLR4 was found in tumor cells, what resulted in downregulation of pro-inflammatory cytokine response (Bussey et al. 2014). Oligoclonal CD8 T cell responses against pulmonary metastatic cancer could be induced by a phospholipid-conjugated TLR7 agonist (Hosoya et al. 2018). In acute retrovirus infection TLR ligand-induced IL-6 may modify antiviral CD8^+^ T cell reactivity (Wu et al. 2015). Detection of viruses by PRRs is limited, because they are practically devoid of any structures that might be sensed as PAMPs. The only way for the innate immune system to trace viruses and to recognize their RNA or DNA is via anti-nucleic acid-mediated PRRs. Viral genomes differ either in their RNA structure (Chiang et al. 2018) or in the location in cell DNA while viral (or tumor) DNA may be traced in cell cytosol, what never happens in normal cell in which DNA is enclosed in nucleus or mitochondria (Iurescia et al. 2018; Li et al. 2017b; Zevini et al. 2017). This has created novel perspectives for management of malignant tumors.

## Viral RNA Sensing by RLRs

Three members of this family have been identified, including RIG-1, melanoma differentiation-associated protein 5 (MDA5) and laboratory of genetics and physiology 2. It has been found that RIG-1 binds preferentially to short double-strand RNAs, while MDA5 senses preferentially long dsRNA (Schlee 2013). The first two (RIG1 and MDA5 possess cytosolic receptors antivirus-derived RNA in cytoplasm. Both have N-terminal caspase activation and recruitment domain, which permits them to interact with mitochondrial adaptor molecule (MAVS). This interaction results in activation of transcription factors IRF (IFN-regulatory factors) and NF-κB that enter cell nucleus and promote activation of several pro-inflammatory cytokine genes (Wu et al. 2017b). Precise RNA targeting and signaling by RIG-1 are biochemically controlled by coordination of RNA and ATP-binding (Fitzgerald et al. 2017). It has been found, that activation of RLRs by oncolytic viruses or synthetic RNA ligands can induce directly cancer cell death, by the production of IFNs and/or by IFNs-independent route, caspase-3-related way involving MAVS and/or IRF3 (El Maadidi et al. 2014). Small RNAs (siRNAs) are able to activate RIG-1. Non-coding nuclear RNA (U1 and U2) translocate to cytoplasm following ionizing radiation therapy. It is associated with favorable course of cancer in patients triggering MDA5 activation by means of dsRNA-poly (I.C.). In ovarian cancer it results in enhanced expression of HLA class I antigens on tumor cells, release of some cytokines, including type I IFN. This process is DC-dependent, because engulfing MDA5-activated tumor cells by DC creates pro-inflammatory milieu manifested by production of cytolytic cytokines and secretion of IFN-γ by NK cells. Thus, RLRs signaling of tumor cells for cancer immunotherapy has several advantages including induction of cell apoptosis anti-tumor immunity via IFN-dependent T and NK cells (Fig. 1). It is of interest that non-malignant cells are resistant to apoptosis via endogenous bcl-xL pathway in contrast to malignant ones (Bhoopathi et al. 2014).

## cGAS-STING Signaling Pathway

**Fig. 2:**
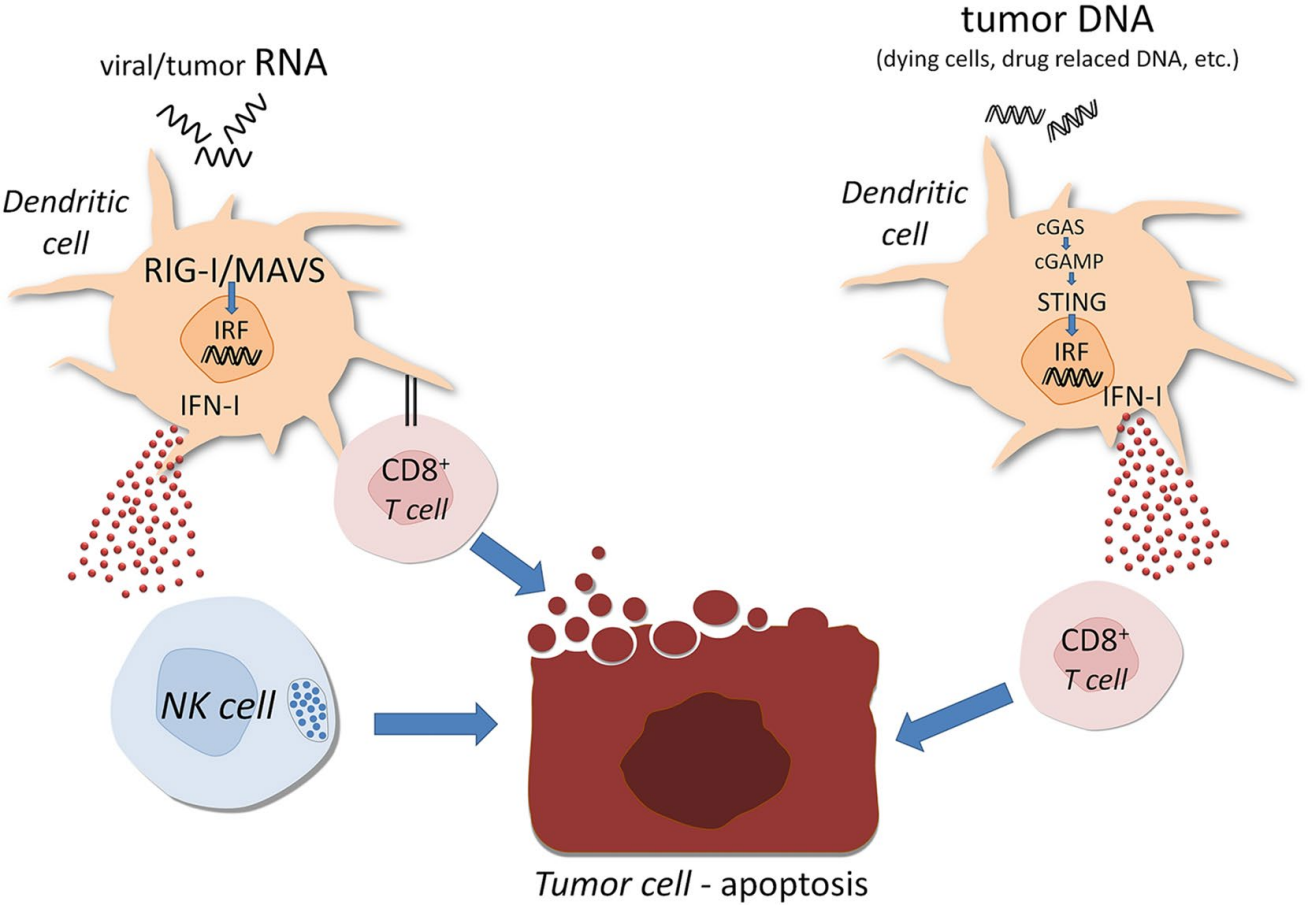
RIG-1 like receptors and STING (stimulator of interferon genes) follow-ups in cancer

This system is based on the recognition of nucleic acids by means of cytosolic DNA sensors. cGMP-AMP synthase (cGAS) synthesized by cGAS from ATP and GTP can directly bind STING (stimulator of interferon genes) following DNA recognition. Practically all pathogens contain and need DNA in their life cycle. There are specific mechanisms permitting to distinguish self from non-self DNA, such as location (pathogenic DNA is found in cytoplasm, while self DNA is present in nucleus and mitochondria). Cytosolic DNA in cancer cells may be derived from nuclear genomic DNA induced by carcinogens and radiation, from micronuclei, from DNA damage due to cell cycle arrest or mutations (Ho et al. 2016). Several DNA sensors have been described, but their significance in tumor pathology is far from clear. In general, activation of STING pathway leads to the production of type I IFNs via IFN-stimulating genes induction and prompt cell death. RIG-1 and STING collaborate, so their activation leads to type I IFN-dependent DC stimulation. The latter drive adaptive immunity via activation of CD8^+^ T cells (Fig. 2). Low STING expression in gastric cancer correlated with tumor size, progression and formation of metastases (Song et al. 2017).

Cancer cells have, however, created means to evade this DNA detection pathway. They include deficient STING translocation to the Golgi apparatus, its normal site, hypermetylation of promoter regions, downregulated cGAS and STING protein expression, how it was shown in colorectal carcinoma and melanoma (Xia et al. 2016a, b). On the other hand, it is known that STING-mediated inflammation may induce tumor initiation, growth and metastatic spread of some cancers, as it was shown in several animal models (Ahn et al. 2015; Lemos et al. 2016).

There are still several doubts whether STING pathway may be applied for the therapy of human tumors (Ng et al. 2018; Sokolowska and Nowis 2018).

## Concluding Remarks

Pattern recognition receptors have been considered for a long time as a biological epiphenomenon without significant importance. It began to change, when their role became evident in the infection and as a bridge between innate and acquired immunity. The detection of PRRs on/in cells of several cancers constituted turning point in shifting interests of scientific community towards these receptors. We know nowadays, that tumor cells exploit in most cases PRRs for their growth and survival. But it also became evident, that we can use man-made agonists to control and inhibit tumor progression. Novel approaches such as GAS-STING signaling pave the way to modern cancer immunotherapy. Nevertheless the statement formulated by Killeen et al. (2006), 13 years ago: Exploitation of the TLR system in cancer: a double-edged sword?—still remains valid, even if our knowledge about PRRs increased dramatically.
