# Correction to: Significance and Role of Pattern Recognition Receptors in Malignancy

**DOI:** 10.1007/s00005-019-00557-2

**Published:** 2019-08-31

**Authors:** Jan Żeromski, Mariusz Kaczmarek, Maciej Boruczkowski, Agata Kierepa, Arleta Kowala-Piaskowska, Iwona Mozer-Lisewska

**Affiliations:** 1 Department of Clinical Immunology, Karol Marcinkowski University of Medical Sciences, Poznań, Poland; 2 Department of Infectious Diseases, Hepatology and Acquired Immunodeficiencies, Karol Marcinkowski University of Medical Sciences, Poznań, Poland

## Correction to: Archivum Immunologiae et Therapiae Experimentalis (2019) 67:133–141 10.1007/s00005-019-00540-x

The authors would like to correct the following error:

In page 139 in Acknowledgements is:

“This work was partly supported by the National Science Center (NCN) under OPUS Grant 2016/23/B/NZ6/013497 (Awarded to prof. IM-L).”

Should be:

“This work was partly supported by the National Science Center (NCN) under OPUS Grant 2016/23/B/NZ6/01497 (Awarded to prof. IM-L).”

